# Mechanical Mechanisms of Chromosome Segregation

**DOI:** 10.3390/cells10020465

**Published:** 2021-02-22

**Authors:** Maya I. Anjur-Dietrich, Colm P. Kelleher, Daniel J. Needleman

**Affiliations:** 1 School of Engineering and Applied Sciences, Harvard University, Cambridge, MA 02138, USA; mayaanjurdietrich@g.harvard.edu; 2 Department of Molecular and Cellular Biology, Harvard University, Cambridge, MA 02138, USA; colmkelleher@fas.harvard.edu; 3 Center for Computational Biology, Flatiron Institute, New York, NY 10010, USA

**Keywords:** chromosome segregation, anaphase, mechanics, spindle

## Abstract

Chromosome segregation—the partitioning of genetic material into two daughter cells—is one of the most crucial processes in cell division. In all Eukaryotes, chromosome segregation is driven by the spindle, a microtubule-based, self-organizing subcellular structure. Extensive research performed over the past 150 years has identified numerous commonalities and contrasts between spindles in different systems. In this review, we use simple coarse-grained models to organize and integrate previous studies of chromosome segregation. We discuss sites of force generation in spindles and fundamental mechanical principles that any understanding of chromosome segregation must be based upon. We argue that conserved sites of force generation may interact differently in different spindles, leading to distinct mechanical mechanisms of chromosome segregation. We suggest experiments to determine which mechanical mechanism is operative in a particular spindle under study. Finally, we propose that combining biophysical experiments, coarse-grained theories, and evolutionary genetics will be a productive approach to enhance our understanding of chromosome segregation in the future.

## 1. Introduction

Cell division—the physical splitting of a mother cell into two daughter cells—is a fundamental and ubiquitous biological process: in the human body, several million cells divide every second [1]. In a successful cell division, each daughter cell inherits a complete copy of the mother cell’s genetic material. This entails duplicating the genetic material in S-phase, then packing it into condensed chromosomes in prophase, and finally segregating the chromosomes into the two daughters in anaphase.

The mechanism of chromosome segregation has long been one of the central mysteries of cell division: what actually causes chromosomes to move apart in anaphase as they do? Anaphase chromosome motions, like all motions, are ultimately caused by forces. Thus, understanding the motions of chromosomes in anaphase requires understanding the forces that cause those motions. In all Eukaryotes, these forces are generated by a self-organized structure called the spindle. The spindle, and the motions of chromosomes during cell division, have been intensely studied for nearly one hundred and fifty years [2]. Despite this extensive work, the manner by which cellular forces produce anaphase chromosome motions is still not well understood [3,4,5,6,7].

In this manuscript, we review prior work on chromosome segregation and discuss our perspective on the mechanics of chromosome motion in anaphase.

It is challenging to organize and integrate the massive amount of information that has been obtained on chromosome segregation. One of the greatest difficulties is the tension between emphasizing commonalities or contrasts when considering different spindles. From one perspective, all spindles have a great deal in common: they are primarily composed of dynamic polymers called microtubules, which rapidly grow and shrink; the nucleation and polymerization of microtubules are modulated in space and time, which aids in accurate chromosome segregation; molecular motors cross-link microtubules and induce their relative sliding, which is key to the organization and assembly of the spindle; and chromosomes attach to the microtubules of the spindle via a complex structure called the kinetochore, which transduces forces from dynamic microtubules. From another perspective, there is great diversity between spindles: while many proteins that make up the spindle are highly conserved, no mitotic molecular motors are universally conserved [8,9]; and the composition and architecture of kinetochores can exhibit large differences between species [10,11,12]. Some spindles contain specialized structures at their poles, called centrioles, which are often at the center of large astral arrays of microtubules, while other spindles lack these [7,13]; the spindle forms inside the nucleus in some cells, while in other cells, the nuclear envelope breaks down and the spindle forms in the cytoplasm [14]. In some spindles, chromosomes move closer to the poles as they separate (termed anaphase A) [15], while in other spindles, poles move apart during chromosomes segregation (termed anaphase B) [16]. In many spindles, both anaphase A and anaphase B occur simultaneously [7].

How to properly weigh these commonalities and contrasts when considering the mechanics of chromosome motion in anaphase? One extreme approach is to attempt to develop a theory of the mechanics of an imaginary, universal spindle that is a weighted average of the properties of different spindles. However, since no such Platonic spindle actually exists, the resulting theory could never be tested. Furthermore, the mechanism of chromosome segregation might really be different in different spindles, making it fallacious to attempt to develop just one model of “The Spindle”. The opposite extreme is to insist that every spindle in every organism must be studied and understood in isolation. However, such a myopic view would cause many fascinating scientific questions to be neglected. For example, in *Xenopus* egg extract spindles, dynein is the molecular motor that most significantly contributes to pole formation [17], whereas kinesin-14 plays a central role in this process in *Drosophila* S2 cells [18]. What about dynein and kinesin-14 allows them to both act as pole-focusing motors? The detailed structure and mechanochemistry of those two motors are quite different; so, clearly, the answer does not lie at the molecular level. Thus, understanding what makes a pole-focusing motor a pole-focusing motor requires thinking about molecular motors, and the spindle, at a more coarse-grained level. Coarse-grained descriptions are also strongly connected to the concept of systems drift: that a developmental or cellular phenotype can remain static even as the mechanisms responsible for that phenotype change over evolution [19]. To state that different phenotypes can be the same even if they result from different mechanisms requires describing the phenotype on a coarse-grained level. This suggests that, for example, the relevant forces acting on chromosomes in anaphase (the cellular phenotype) may be the same in different spindles, even if the origin of those forces (the molecular and biophysical mechanisms) differs. Understanding the extent to which this is true will, of course, require understanding the forces acting on chromosomes in different spindles.

In this manuscript, we argue that describing the spindle at a coarse-grained level provides a powerful means to address both commonalities and contrasts in the mechanics of chromosome motion in anaphase. Such an approach lets us emphasize cellular-scale processes, which is the natural level of description for the micron-scale movements of chromosomes that occur during anaphase. A coarse-grained cellular-scale resolution also corresponds to the resolution with which we typically experimentally study anaphase chromosome motions, i.e., the resolution of the light microscope. Matching our theoretical resolution to our experimental resolution can aid comprehension, and helps ensure that we construct theories that are amenable to being experimentally tested.

In the rest of this review, we will use this coarse-grained perspective to examine the sites of force generation in spindles, how those sites can be mechanically coupled to each other and to chromosomes, and the manner in which these forces and couplings can lead to chromosome segregation. There are believed to be three primary locations where relevant forces are generated: the astral regions, the kinetochore, and the so-called central spindle that sits between segregating chromosomes. In Section 2, we describe these sites of force generation. Knowing the sites of force generation is not sufficient to determine which forces are responsible for chromosome motion; in Section 3, we review relevant concepts from mechanics and their application to chromosome motion to explain why this is so. In Section 4, we combine the results from the previous two sections to propose different mechanical mechanisms of chromosome segregation that might operate in different spindles. We suggest experiments that can be performed to test which mechanical mechanisms are operative in particular spindles under study. We conclude in Section 5 by discussing possible future directions. We argue that truly understanding the commonalities and contrasts in chromosome segregation in different spindles will require integrating such mechanistic models into an evolutionary framework.

## 2. Force Generation in Anaphase Spindles

Direct measurements of forces on segregating chromosomes in cells were performed approximately forty years ago by R.B. Nicklas [20,21]. He found that grasshopper spermatocytes spindles are capable of exerting forces as high as ~700 pN on chromosomes. As we will describe in more detail in Section 3, spindles likely generate much smaller forces during the normal course of chromosome segregation. It might seem reasonable to conclude that these forces from the spindle are generated by kinetochores since it is known that kinetochores apply forces to chromosomes. In Section 3 and Section 4, we will explain that the validity of that interpretation depends on what other sites of force generation are present in the spindle, and how those sites are mechanically coupled to each other and to chromosomes.

**Figure 1:**
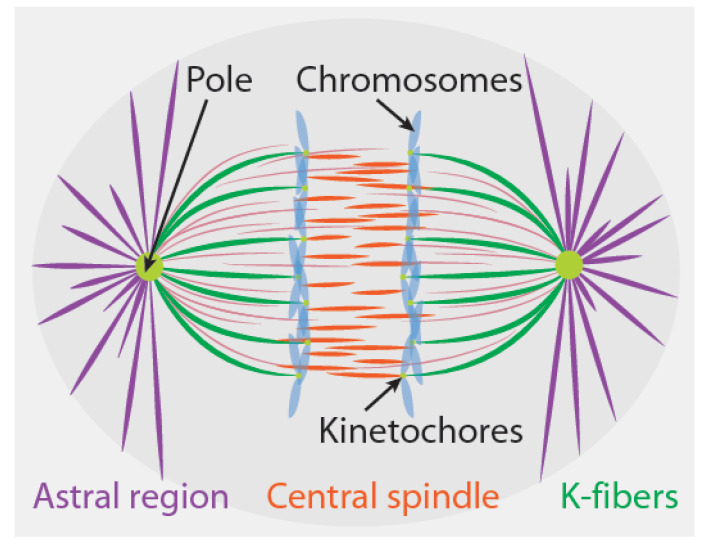
Three force-generating sites that are found in diverse spindles are kinetochores, the astral region, and the central spindle.

Different sites of force generation have been proposed to contribute to chromosome segregation in different cell types [3,4,5,6,7]. In this section, we briefly review three sites of force generation that have been particularly widely discussed: the kinetochore, the astral regions, and the central spindle (Figure 1). From an abstract perspective, one can envision different spindles as being composed from different combinations of these same three fundamental force-generating “modules”. It is believed that only one or two of these sites produce forces in some spindles, while in other spindles, all three of these sites are active. We limit our discussion to these three sites of force generation in spindles for simplicity, even though others have also been proposed (see Section 4.2). As we will elaborate on further in Section 3, Section 4 and Section 5, even when these three sites of force generation are all present in a spindle, the extent to which they each contribute to chromosome segregation depends on how they interact with each other and with chromosomes.

### 2.1. Kinetochores

Kinetochores are both signaling hubs and sites of force generation in mitosis (Figure 1) [22,23]. In the region between poles and chromosomes, the majority of microtubules have their plus ends pointed towards the chromosomes and their minus ends towards the pole; if the plus end is embedded in the kinetochore, that microtubule is referred to as a kinetochore microtubule. The collection of all microtubules attached to a single kinetochore is called a kinetochore fiber (or a K-fiber). Metazoan K-fibers consist of bundles of ~5–40 microtubules [24,25,26,27,28]. While some kinetochore microtubules extend all the way to the pole, others do not, and the relative fraction of pole-associated kinetochore microtubules varies between different spindles [24,25,29].

During anaphase, in many spindles, but not all [30], kinetochore microtubules depolymerize from their plus ends while remaining attached to the kinetochore [15,31]. Such a behavior requires relative motion between kinetochore microtubules and the kinetochore, and strongly implies that the kinetochore is a site of active force generation. A purified yeast kinetochore attached to a single depolymerizing microtubule can exert forces in excess of 10 pN [32]. If the force scales linearly with the number of kinetochore microtubules, then Metazoan kinetochores should be able to produce many hundreds of pNs. The precise biophysical mechanism by which depolymerizing microtubules produce forces at the kinetochore is still unclear, and a variety of different models have been proposed [33,34,35,36].

### 2.2. The Astral Region

Radial arrays of microtubules surround the poles of many, but not all, spindles (Figure 1) [7,13]. These so-called astral microtubules point with their plus ends emanating outward, away from the pole [37,38]. When astral microtubules are present, they often play a key role in generating the forces that position the spindle [39,40,41,42]. The precise nature of the forces exerted by astral microtubules to position the spindle remains controversial. Some researchers favor models in which pulling forces stably position the spindle, while others favor models where pulling forces are destabilizing and pushing forces are stabilizing. Disagreement on this point even exists regarding the nature of forces acting on the first mitotic spindle in *Caenorhabditis elegans* (*C. elegans*) embryos [43,44], which is arguably the system where this issue has been most thoroughly studied. In addition to their role in spindle positioning, extensive evidence in diverse systems also shows that pulling forces from astral microtubules drives spindle elongation [16]. The contribution of these forces has also been most intensely studied in the first mitotic division of *C. elegans*. In that system, the spindle poles are stably positioned by forces exerted from astral microtubules, both before the start of spindle elongation and after spindle elongation has ceased [43,44]. Thus, spindle elongation in *C. elegans* entails the poles switching from one initially stable position to a second, final stable position. This change is likely driven by the increase in astral pulling forces that occurs at the onset of anaphase [45,46]. In some spindles, microtubules depolymerize from their minus ends at poles, resulting in a relative velocity between the microtubules and poles [7,16]. Such depolymerization at poles is expected to result in active force generation, which is analogous to the microtubule depolymerization-based force at kinetochores.

### 2.3. The Central Spindle

At anaphase onset, a new region of microtubules is established in the central spindle, between the separating sister chromatids (Figure 1). The plus ends of the central spindle microtubules overlap in the middle of this region. Extensive evidence shows that pushing forces from the central spindle drives spindle elongation in diverse systems [7,16]. In smaller spindles, such as those from yeasts [47,48], microtubules from the central spindle extend to the poles. Such half-spindle-spanning microtubules appear to be absent in larger, metazoan spindles [49,50], which contain many microtubules with both of their ends between segregating chromosomes. Central spindle microtubules simultaneously elongate and slide relative to each other during anaphase. As far as we are aware, there are no published direct measurements of the force produced by the central spindle, but, in spindles undergoing closed mitosis, this force can be strong enough to distort the nucleus [51] or cause the spindle to buckle [52]. The central spindle contains passive cross-linkers, which can produce both entropic forces [53] and frictional forces [54] between microtubules. It is still unclear how these passive cross-linkers interact with molecular motors and microtubule polymerization dynamics to organize the central spindle and generate forces [55,56,57].

## 3. Mechanics and Its Relevance to Anaphase Spindles

Motions result from forces, but the manner in which that occurs can be surprisingly subtle and counterintuitive. In this section, we review aspects of mechanics that are relevant for understanding chromosome segregation. We present simple, coarse-grained models for the behaviors of force generators in the spindle. One of the central conclusions of this discussion is that knowing that a site of force generation is present in a spindle is not sufficient to determine if the forces produced by that site contribute to chromosome motion. This point will be further elaborated on in Section 4, where we will argue that the same three sites of force generation (described in Section 2) can be connected in different ways to produce mechanically distinct mechanisms of chromosome segregation.

### 3.1. Force Balance

It seems natural to ask, “what is the force acting on a chromosome in anaphase?” Taken literally, the answer to this question is trivial: the total force acting on a chromosome is zero. The reason is that chromosomes, like all other structures in cells, are small objects that move slowly in an environment that is permeated by a viscous fluid. This means that they exist at low Reynolds number, a regime in which inertia is negligible relative to viscosity [58,59]. From Newton’s second law, $F_{total}=ma$, where $F_{total}$ is the total force acting on the chromosome, a is the acceleration of the chromosome, and m is the mass of the chromosome. Because inertia is insignificant at low Reynolds number, it is a very good approximation to treat ma as being zero, giving $F_{total}=0$.

Since the total force on a chromosome is zero, all of the separate forces acting on it must balance. It is this balance of forces that ultimately drives chromosome segregation. But what are the different forces that act on chromosomes, and how do they produce chromosome motion in anaphase?

### 3.2. The Relevance, or Lack Thereof, of Fluid Drag Forces

**Figure 2:**
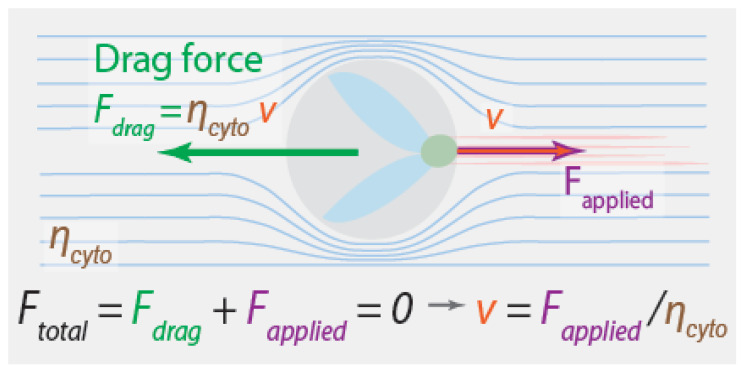
The motion of an isolated chromosome dragged by an optical tweezer. The velocity of the chromosome is determined by the balance of the applied force from the optical tweezer, $F_{applied}$, and the fluid drag force, $F_{drag}=-\eta_{cyto}v$.

The forces acting on a chromosome must depend on its velocity, and the velocity it actually moves at must be such that the total force on the chromosome is zero. One natural guess is that the relevant balance of forces is between fluid drag forces and the forces that the spindle applies to the chromosomes: $F_{total}=0=F_{drag}+F_{applied}$ (Figure 2). The drag force is proportional to the difference between the velocity of the chromosome, v, and the velocity of the fluid it is immersed in, $v_{fl}$, giving $F_{drag}=-\eta_{cyto}\left(v-v_{fl}\right)$. We will assume that, far away from the moving chromosomes, the fluid is stationary relative to the boundary of the cell, so $v_{fl}=0$, and v is then also the relative velocity between the chromosome and the cell. $\eta_{cyto}$ is a constant that sets the magnitude of the drag force, and depends on the size and shape of the chromosomes and the viscosity of the cytoplasm [60]. Taken together, this leads to a formula for the velocity of a chromosome:

(1) $$v=\frac{F_{applied}}{\eta_{cyto}}$$

An equation like this is a valid coarse-grained description for an isolated chromosome being dragged through an aqueous solution by application of forces from an optical tweezer. But is it relevant for understanding what drives chromosome motions in spindles?

One hint comes from the observation that, in many systems, all chromosomes move at the same speed during anaphase, even though their size varies by at least a factor of two [61]. This is surprising, because doubling the size of a chromosome also doubles the drag force on it, which, from Equation (1), would halve the velocity for the same applied force. The observed constant velocity for chromosomes of different sizes implies that the forces produced by the spindle are dependent on the forces exerted on it. Nicklas used this observation to argue that the spindle contains a “speed governor”: instead of producing a constant force, the spindle adjusts the force that it produces to set the speed of the chromosomes. This implies that fluid drag forces are not relevant for typical anaphase chromosome motions.

A similar conclusion results from considering the expected magnitude of drag forces on chromosomes. Plugging in reasonable numbers for the size of the chromosomes and the viscosity of the cytoplasm, predicts $\eta_{cyto}\approx 0.005\;pN\;s/nm$ [61]. For a chromosome moving at v≈1 μm/min, as chromosomes in grasshopper spermatocytes do, this gives $F_{drag}\approx 0.1\;pN$. This calculated drag force is roughly ten thousand times less than the maximum force that spindles in grasshopper spermatocytes can produce, as measured by Nicklas [20,21]. This provides further evidence that the spindle contains a “speed governor”. It also argues that the spindle is so overpowered that fluid drag forces are irrelevant for chromosome segregation.

A somewhat related point has recently been made regarding heavily cross-linked networks of molecular motors and microtubules. An in vitro experiment on large aligned arrays of microtubules and kinesin-14 found that microtubules slide apart from each other at a speed that is independent of the local polarity of the network (i.e., the number of left vs. right facing microtubules), which would not occur if fluid drag forces on microtubules are significant [62,63]. A similar independence of microtubule sliding speed and polarity is seen in *Xenopus* egg extract spindles, arguing that fluid drag is also likely irrelevant for microtubule motions in those spindles. This further supports the contention that fluid drag is likely not significant for the internal dynamics of spindles, including anaphase chromosome motions.

### 3.3. Mechanics of Speed Governors

**Figure 3:**
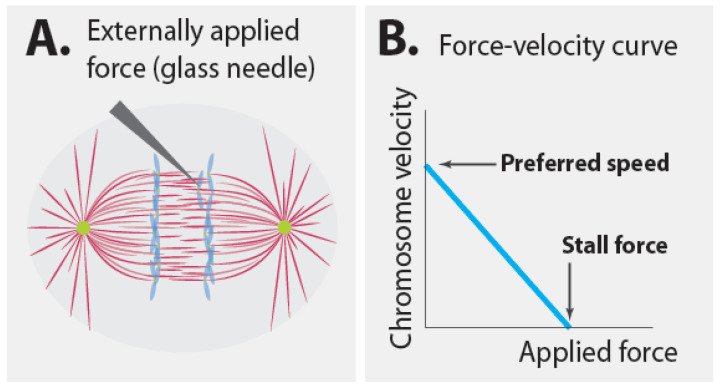
(**A**) Nicklas used a glass needle to apply force on anaphase chromosomes and slowed their motion. (**B**) He characterized the relationship between the applied force and velocity using a force–velocity curve. When no force is applied, the chromosomes move at the spindle’s preferred speed. Since the spindle has a preferred speed, it contains a speed governor. The stall force is the applied force that causes the chromosomes to stop moving. The origin of the speed governor in the spindle remains unclear, and, as will be elaborated on in Section 4, it might not reflect the force-generating properties of the kinetochore.

It seems clear that the speed of chromosome motion in anaphase is set by a speed governor. The presence of a speed governor has strong implications for the mechanism of chromosome segregation, as will be elaborated in more detail below. However, the mechanics of speed governors can be quite counterintuitive. Consider Nicklas’ classic measurement in more detail [20]. Nicklas used a calibrated glass needle to apply known forces to resist the motion of segregating chromosomes in anaphase (Figure 3A). In the absence of an applied force, the chromosomes moved at their preferred speed. The greater the force that Nicklas applied, the slower the chromosomes moved. Thus, there is a relationship between applied force and velocity, which can be represented by a force–velocity curve (Figure 3B). Since the total force acting on the chromosome is always zero (because of force balance), when Nicklas applied a higher force to the chromosome with his glass needle, the spindle must have applied a correspondingly higher force in return to keep the chromosome in motion. When the applied force was high enough, ~700 pN, the chromosomes ceased to move. This force, i.e., the applied force necessary to stop all motion, is known as the stall force. Thus, the spindle can exert up to ~700 pN on a single chromosome. What does this experiment say about the magnitude of force that the spindle exerts on a chromosome during unperturbed anaphase (i.e., in the absence of additional forces from the glass needle)? Absolutely nothing. Instead, this experiment informs us about the additional forces that the spindle can produce beyond those that it normally does. Hypothetically, the force that the spindle normally produces could be determined by measuring the tension in all load-bearing linkages in an unperturbed spindle [64,65,66], but this is a fundamentally different question than that addressed by Nicklas’s experiment.

The mechanistic basis of the spindle’s speed governor is still unclear. As we will discuss in detail in Section 4, different mechanical models of chromosome segregation result in different predictions for the origin of the forces that move chromosomes. Still, some intuition can be gained by thinking of individual molecular motors or polymerizing microtubules, which contain their own speed governors and exhibit approximately linear-force velocity curves [67]. Consider an individual molecular motor walking on a microtubule. A molecular motor is an enzyme that hydrolyzes ATP (a reaction that, of course, is coupled to motion). Just like any enzyme, the rate of the reaction that it catalyzes depends on the rates of the conformational changes that it goes through. For molecular motors, the rates of some of these conformational changes are impacted by applied forces. Thus, the speed of the hydrolysis reaction, and hence the speed that the motor walks at, depends on the applied force. The molecular motor’s preferred speed is set by the rate that it hydrolyzes ATP in the absence of an applied force, and the molecular motor’s force–velocity curve describes how it changes its speed in response to an applied force. In a similar vein, the spindle’s speed governor is presumably a force-dependent enzymatic reaction that is coupled to chromosome motion and is likely caused by some combination of molecular motors, microtubule polymerization, and microtubule depolymerization.

As described above, a system with a speed governor, such as the spindle, adjusts the amount of force it produces depending on the force applied to it. The actual speed that a chromosome in the spindle moves at will be given by where this applied force causes it to fall on the spindle’s force-velocity curve. If the only forces acting on the chromosome are from the spindle and fluid drag, then those two forces must be equal and opposite (by force balance). If fluid drag is sufficiently small, then the spindle will operate very close to its preferred speed. In this scenario, the force from fluid drag does not significantly impact chromosome motion, even though it has the same magnitude as the force from the spindle. Thus, the spindle’s force-velocity curve provides a simple explanation for the apparent irrelevance of fluid drag for anaphase chromosome motions.

### 3.4. Forces Are Local and Dependent on Relative Positions and Velocities

The spindle contains a speed governor that modulates the force it exerts on chromosomes. But how does this force result from the force generators discussed in Section 2? How do the force generators in the spindle produce chromosome motion in anaphase? When contemplating these issues, it is crucial to remember that forces relevant to cell biology are extremely short ranged, typically extending nanometers or less [68]. Therefore, cell biological forces result from objects locally pushing and pulling each other. The forces acting between objects can only depend on their immediate environment and, by Newton’s third law, they must be equal and opposite. We next explore the implications of these facts for forces generated from the three sites discussed in Section 2.

#### 3.4.1. Implications for Forces from Kinetochores

**Figure 4:**
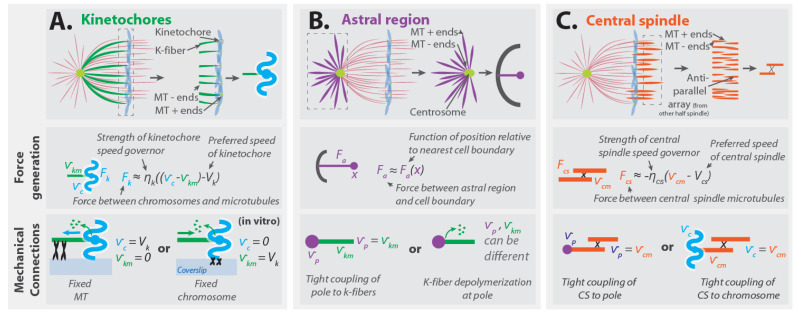
((**A**), top) A coarse-grained mechanical model of kinetochore forces and K-fibers, which are bundles composed of kinetochore microtubules. ((**A**), middle) Kinetochores apply forces between chromosomes and kinetochore microtubules, $F_{k}$. This force depends on the extent to which the relative velocity of chromosomes, $v_{c}$, and kinetochore microtubules, $v_{km}$, differs from the kinetochore’s preferred speed, $V_{k}$. ((**A**), bottom) In vitro movement of the microtubules and chromosomes is dependent on which mechanical linkages are present. ((**B**), top) A coarse-grained mechanical model of the astral region forces. ((**B**), middle) The astral region applies forces between the pole and the cell boundary, $F_{a}$. This force depends on the distance between the pole and the cell boundary, x. ((**B**), bottom) Two different possible couplings with implications for the relationship between the velocity of kinetochore microtubules, $v_{km}$, and the velocity of spindle poles, $v_{p}$. ((**C**), top) A coarse-grained mechanical model of central spindle forces. ((**C**), middle) The central spindle applies a force between the central spindle microtubules, $F_{cs}$. This force depends on the extent to which the velocity of the central spindle microtubules, $v_{cm}$, differs from the central spindle’s preferred speed, $V_{cs}$. ((**C**), bottom) Two different possible couplings of central spindle microtubules: either tightly coupled to chromosomes or tightly coupled to poles.

Consider depolymerizing microtubules whose plus ends are embedded in a kinetochore (Figure 4A, top). Since the microtubules remain attached to the kinetochore as they depolymerize, there must be a velocity difference between the microtubules and the chromosome that the kinetochore is part of. The force that the kinetochore exerts will depend on the difference between the velocity of the chromosome, $v_{c}$, and the velocity of the kinetochore microtubules, $v_{km}$. Note that $v_{km}$ is the velocity of the tubulin monomers in the microtubules, which can be different from the center of mass velocity of these microtubules if they polymerize or depolymerize. The kinetochore contains a “speed governor” with a preferred speed, $V_{k}$, and a force must be applied to alter the kinetochore’s speed away from this value [32]. Thus, the force that the kinetochore generates, $F_{k}$, will depend on the extent to which the velocity difference between the chromosome and microtubules deviates from the preferred speed of the kinetochore. If the velocity difference between the kinetochore microtubules and the chromosome is close to the kinetochore’s preferred speed, the force–velocity relationship can be approximately described as linear:

(2) $$F_{k}\approx -\eta_{k}\left(\left(v_{c}-v_{km}\right)-V_{k}\right)$$

where $\eta_{k}$ is a constant that characterizes the “strength” of the force-generating machinery in the kinetochore in this coarse-grained description (Figure 4A, middle). The force that the kinetochore exerts on the kinetochore microtubules will be equal and opposite to the force that the kinetochore exerts on the chromosome. If this linear description is valid for the entire force–velocity curve, not just when the velocity difference is close to the preferred speed, then the kinetochores stall force is $\eta_{k}V_{k}$ (i.e., if $F_{k}=\eta_{k}V_{k}$, then $v_{c}-v_{km}=0$).

Since the force generated by the kinetochore is based on its local interactions with microtubules, this force influences the relative motion of the microtubules and chromosome but is not sufficient to determine how the chromosomes will actually move. Consider an in vitro experiment in which chromosomes are subjected to kinetochore-generated forces due to their interactions with microtubules, with the minus ends of the microtubules rigidly attached to a coverslip [69,70]. Since the microtubules are fixed to the coverslip, they will be stationary (with respect to the coverslip), while the chromosome will move relative to the coverslip (Figure 4A, bottom left). Alternatively, if the chromosome was fixed to the coverslip and the microtubules were free to move, then the chromosomes would remain stationary (relative to the coverslip), while the microtubules would be reeled in by the kinetochore (Figure 4A, bottom right). Thus, the forces from the kinetochore induce relative motion between the kinetochore microtubules and chromosomes, and the extent to which these impact the movement of chromosomes (relative to the coverslip or the cell) depends on what other forces are present.

If the kinetochore microtubules are held stationary and the only forces acting on the chromosome are from the kinetochore and drag, then $F_{drag}+F_{k}=0$, and combining Equations (1) and (2) gives $v_{c}=\frac{\eta_{k}}{\eta_{cyto}+\eta_{k}}V_{k}$. As mentioned above, $\eta_{cyto}\approx 0.005\;pN\;s/nm$ for metazoan chromosomes. Extrapolating from Akiyoshi et al.’s data, with the assumption that $\eta_{k}$ scales linearly with the number of kinetochore microtubules, gives $\eta_{k}\approx 0.1\;pN\;s/nm$ for metazoan kinetochores. Using these numbers results in $v_{c}\approx 0.95\;V_{k}$. Since under this scenario the chromosome’s velocity is 95% of the kinetochore’s preferred speed, it is quite reasonable to neglect fluid drag entirely, which leads to $v_{c}=V_{k}$. Note that, in this approximation, the relative velocity between the chromosome and the microtubules is the kinetochore’s preferred speed, $v_{c}-v_{km}=V_{k}$. Thus, from Equation (2), the force that the kinetochore exerts will be zero. So, surprisingly, this results in a force-balance model that explains chromosome motion in which all of the individual forces are well-approximated as being zero. While this might seem paradoxical, it is a consequence of basic mechanics. It also highlights that, for understanding motions, the functional form of forces can be more important than their magnitudes.

#### 3.4.2. Implications for Forces from the Astral Region

Forces from the astral region can both position the spindle and drive its elongation. While the same forces may be responsible for both these processes, their contribution to spindle positioning has been investigated in greater detail. The forces from the astral region occur due to interactions with the cell boundaries (Figure 4B, top). Since these forces can stably position the spindle, the net force from the astral regions acting on the spindle, $F_{a,s}$, will depend on the extent to which the location of the spindle, $x_{s}$, deviates from its equilibrium position in the cell, $X_{a,s}$. When the spindle is at its equilibrium position, the net force from the astral regions is zero (by force balance), so $F_{a,s}\left(0\right)=0$. The simplest coarse-grained description is one in which this force acts as a linear spring: $F_{a,s}\approx k\left(x_{s}-X_{a,s}\right)$, where k is a spring constant that characterizes the strength of forces from the astral region. Measurements of forces on the astral region of spindles in cells have only been reported in one published manuscript, in which the authors interpreted their measurements using a linear spring-based model ([44], for closely related work, also see [71]). This work, by Garzon-Coral et al., found spring constants associated with displacing spindle poles in the first mitotic division of *C. elegans* to be of order 15 pN/μm in metaphase and of order 90 pN/μm in anaphase.

The stable positioning of the spindle from astral forces is due to a balance of equal and opposite forces acting on both poles: $F_{a,s}=F_{a,p1}+F_{a,p2}$, where $F_{a,p1}$ and $F_{a,p2}$ are the astral forces acting on the two poles, located at distances $x_{p,1}$ and $x_{p,2}$ on opposite sides of the cell center. If the spindle is stably positioned in the center of the cell and the forces on the two centrosomes are equivalent, then $X_{a,s}=0$, $F_{a,p1}=F_{a}\left(x_{p,1}\right)$, and $F_{a,p1}=-F_{a}\left(x_{p,2}\right)$, where $F_{a}\left(x\right)$ characterizes the force the astral microtubules exert on a pole as a function of its position relative to the nearest cell boundary (Figure 4B, middle). Hypothetically, the astral region might also contain a speed governor, but, for simplicity, we will not consider that in this manuscript.

A very simple possibility is that when the spindle is stably positioned with a constant length, each pole is stationary because the astral force acting on it is balanced by the tension in the spindle (though such a description is not always appropriate [43]). In this case, if one of the poles were disconnected from the rest of the spindle (by a laser ablation experiment, for example), the astral force on the pole would no longer be balanced by tension in the spindle [46]. Instead, the pole would experience a drag force equal in magnitude to the astral force, and its motion would be governed by an equation analogous to Equation (1), leading it to move towards the cell membrane with a velocity of $v=\frac{F_{a}}{\eta_{cyto}}$. The detached pole would be expected to move quickly even if the magnitude of astral forces is small, because, as discussed above, the cytoplasmic drag is so small. Thus, cytoplasmic drag forces may be crucial for understanding the motion of spindle fragments, or the entire spindle, even if they do not significantly influence anaphase chromosome motions.

Since forces from astral microtubules are applied locally to the pole, the impact of these forces on chromosome motion depends on the manner by which the pole and chromosomes are connected. One important type of coupling is through kinetochore microtubules that bind to both structures. If one naively neglects deformations and (de)polymerization at the microtubule minus ends, such a coupling would imply that the poles and kinetochore microtubules move together: i.e., that the velocity of the poles, $v_{p}$, is the same as the velocity of the kinetochore microtubules, $v_{km}$, giving $v_{p}=v_{km}$ (Figure 4B, bottom left). Connections between the pole and kinetochore microtubules can also be indirect, through cross-linking with non-kinetochore microtubules [29,72,73]. A coupling similar to Equation (2) is expected if kinetochore microtubule minus ends actively depolymerize at poles [74], which will generally result in kinetochore microtubules and poles having different velocities (Figure 4B, bottom right). The movement of the poles themselves also depends on the nature of their connections and the strength of the forces acting on them. In spindles in which the astral forces are large enough, they can cause the two poles to move relative to each other, or relative to the chromosomes, thereby elongating the spindle. In spindles in which astral forces are very weak, these forces may stably position the spindle without significantly impacting spindle elongation or chromosome segregation.

#### 3.4.3. Implications for Forces from the Central Spindle

Forces from the central spindle are believed to be locally generated in the region of anti-parallel microtubule overlap (Figure 4C, top) [7,16,75]. These forces cause the central spindle microtubules of opposite polarity to slide apart from each other as they simultaneously grow longer. Spindles often contain more central spindle microtubules than kinetochore microtubules; so, if these two regions produce a similar amount of force per microtubule, then the central spindle may be able to generate greater forces than kinetochores [25,29,47,48,49,50]. Thus, though it has not yet been experimentally demonstrated, the relative sliding of central spindle microtubules is presumably set by an overpowered “speed governor”. This implies that the force between central spindle microtubules follows a force-velocity relationship analogous to Equation (2): $F_{cs}\approx -\frac{\eta_{cs}}{2}\left(\left(v_{cm,1}-v_{cm,2}\right)-2V_{cs}\right)$, where $\eta_{cs}$ characterizes the strengthen of this force-generating machinery, $v_{cm,1}$ and $v_{cm,2}$ are the velocities of central spindle microtubules facing in the two different directions, and 2$V_{cs}$ is the preferred speed for the central spindle microtubules to slide away from each other (Figure 4C, middle). Under the reasonable assumption that the two halves of the spindle behave equivalently (and thus $v_{cm,1}$ = $-v_{cm,2}=v_{cm}$) this becomes

(3) $$F_{cs}\approx -\eta_{cs}\left(v_{cm}-V_{cs}\right)$$

Note that $v_{cm}$ is the velocity of tubulin monomers in the central spindle microtubules, which can be different from the center of mass velocity of these microtubules if they polymerize or depolymerize.

The manner and extent to which central spindle forces impact chromosome segregation depends on how the central spindle is connected to chromosomes. In smaller spindles, such as those in yeasts, central spindle microtubules are believed to primarily couple to spindle poles [7]. Such a tight connection naively suggest that the velocity of the poles, $v_{p}$, is the same as the velocity of the central spindle microtubules, $v_{cm}$, giving $v_{p}=v_{cm}$ (Figure 4C, bottom right). This implies that the influence of the central spindle on chromosomes is indirect, and only occurs through their mutual interactions with the spindle pole. In larger, metazoan spindles, micromanipulation [76], laser ablation [77,78,79,80], and structural studies [25,29,49] indicate that non-kinetochore microtubules strongly interact with kinetochore microtubules throughout their length, including very close to the kinetochore itself. In those spindles, it may be more accurate to approximate the central spindle microtubules as being linked directly with the kinetochore (or chromosomes) than as being connected to the pole. This scenario implies that the velocity of the chromosomes, $v_{c}$, is the same as the velocity of the central spindle microtubules, $v_{cm}$, giving $v_{c}=v_{cm}$ (Figure 4C, bottom left).

## 4. Mechanics of Chromosome Segregation

Chromosome segregation is the process in which sister chromatids move away from each other. There are no long-range forces between chromatids that induce their relative motion. Rather, the separation of chromosomes is due to the cumulative effect of local forces causing relative motions of the other components of the spindle: relative motions between chromatids and kinetochore microtubules; between kinetochore microtubules and spindles poles; between the astral microtubules of spindle poles and the cell boundaries; between anti-parallel central spindle microtubules; and between central spindle microtubules and poles or chromosomes. There are different ways that these relative motions can induce chromosome movement in anaphase. Thus, there are different possible mechanical mechanisms of chromosome segregation.

### 4.1. Four Mechanical Models of Anaphase

To illustrate some of the possible mechanical mechanisms of chromosome segregation, we next discuss four different coarse-grained models of anaphase in detail, as well as experiments to determine which may be relevant in different spindles. These models all contain the same three force-generating regions referred to throughout this manuscript—the kinetochore, the astral region, and the central spindle. The models differ in how these force generators are connected and interact, resulting in different mechanisms of anaphase.

We construct models for a spindle simultaneously undergoing anaphase A and anaphase B; i.e., a spindle whose poles and chromatids are moving away from the cell center at velocities $v_{p}$ and $v_{c}$, respectively, with $v_{c}>v_{p}$, such that the chromatids move closer to the poles as the poles separate. We consider spindles that lack microtubule minus end depolymerization at the pole, though it would be straightforward to incorporate that. For simplicity, in some of the models we will approximate the kinetochore or the central spindle to be so dominant that they operate at their preferred speed (denoted as $V_{k}$ for the kinetochore and $V_{cs}$ for the central spindle), while approximating other force generators as being so miniscule that they can be treated as passive mechanical elements or entirely disregarded. We neglect the contribution of fluid drag to internal motions of the spindle, since, as discussed above, its impact is likely very minor (but fluid drag may be relevant for movement of the entire spindle, or detached fragments of the spindle). We assume that the astral region is always generating pulling forces; though, in some models, those forces may not be strong enough to impact the internal structure of the spindle, and thus only act to stably position the spindle in the center of the cell. For simplicity, we approximate the spindle as being one dimensional, meaning that all forces and motions occur along a straight line connecting the poles [74,80,81]. For each model, we calculate the speed of the poles, $v_{p}$, chromatids, $v_{c}$, kinetochore microtubules, $v_{km}$, and central spindle microtubules, $v_{cm}$.

#### 4.1.1. Model 1: Kinetochore- and Central Spindle-Dominated, Pole-Linked

**Figure 5:**
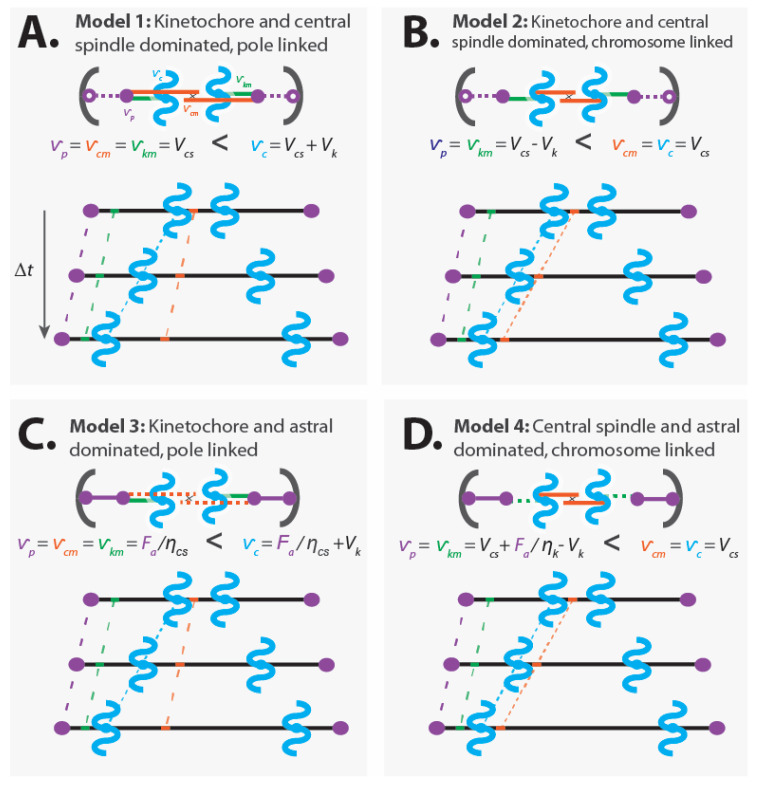
(**A**) In Model 1, the central spindle microtubules slide apart at their preferred speed, $V_{cs}$, the kinetochore causes the kinetochore microtubules and chromatids to move relative to each other at its preferred speed, $V_{k}$, and the central spindle microtubules are rigidly attached to the spindle poles. In this model, the speed that chromosomes move, $v_{c}$, is faster than the speed that central spindle microtubules move, $v_{cm}$ (which is equal to the speed of spindle poles, $v_{p}$, and kinetochore microtubules, $v_{km}$). (**B**) In Model 2, the central spindle microtubules slide apart at their preferred speed, the kinetochore causes the kinetochore microtubules and chromatids to move relative to each other at its preferred speed, and the central spindle microtubules are rigidly attached to the chromatids. In this model, chromosomes move at the same speed as central spindle microtubules. (**C**) In Model 3, the astral region generates substantial forces, $F_{a}$, that pull the spindles poles apart with speed $\frac{F_{a}}{\eta_{cs}}$ (where $\eta_{cs}$ is a measure of the strength of frictional forces between central spindle microtubules), and the central spindle microtubules are rigidly attached to the pole and passively respond to the forces acting on them. The kinetochore causes the kinetochore microtubules and chromatids to move relative to each other at its preferred speed. In this model, chromosomes move faster than the central spindle microtubules. (**D**) In Model 4, the astral region generates substantial forces that are sufficient to perturb the dynamics of the kinetochore (where $\eta_{k}$ is a measure of the strength of kinetochore force generation), while the central spindle microtubules slide apart at their preferred speed. The central spindle microtubules are rigidly attached to the chromatids. In this model, chromosomes move at the same speed as the central spindle microtubules.

Model 1: The central spindle microtubules slide apart at their preferred speed, the kinetochore causes the kinetochore microtubules and chromatids to move relative to each other at its preferred speed, and the central spindle microtubules are rigidly attached to the spindle poles (Figure 5A).

Assumptions:

- Dominant kinetochores: $v_{c}-v_{km}=V_{k}$ (from Equation (2))
- Dominant central spindle: $v_{cm}=V_{cs}$ (from Equation (3))
- Central spindle microtubules are linked to the pole: $v_{p}=v_{cm}$
- Kinetochore microtubules are linked to the pole: $v_{p}=v_{km}$
- Astral pulling forces position the spindle, but do not alter its internal dynamics

Consequences:

- Pole movement: $v_{p}=V_{cs}$
- Chromatid movement: $v_{c}=V_{cs}+V_{k}$
- Central spindle microtubule movement: $v_{cm}=V_{cs}$
- Kinetochore microtubule movement: $v_{km}=V_{cs}$

In this model, chromosome segregation results from a sum of two processes: the poles being pushed apart by the central spindle (with speed $V_{cs}$) and the chromatids being pulled toward the poles by the kinetochore (with speed $V_{k}$).

Hypotheses similar in spirit to Model 1 have been proposed as models for anaphase in diverse spindles. This model seems particularly plausible for yeast spindles. Central spindle microtubules and kinetochore microtubules directly connect to spindle pole bodies in yeast, arguing for the validity of a pole-linked model for those microtubules [47,48]. If Model 1 is valid then the elongation of the central spindle should not be impacted by removing its connection to spindle poles, but anaphase chromosome motion should require an intact attachment between chromosomes and poles. Laser ablation experiments in Fission yeast seem to be consistent with these predictions [82,83].

Model 1 predicts that chromosomes move faster than central spindle microtubules when anaphase A and anaphase B occur simultaneously (i.e., central spindle microtubules move with speed $V_{cs}$ while chromatids move with speed $V_{cs}+V_{k}$) (Figure 5A). As far as we are aware, no measurements showing this result have been reported for any spindle. Observing such behavior would provide strong additional support for this model. Second, perturbing the central spindle microtubules (i.e., $V_{cs}\to V_{cs}^{\prime }$) is predicted to impact pole motion ($v_{p}\to V_{cs}^{\prime }$) without influencing the relative movement of chromatids and poles ($v_{c}-v_{p}=V_{k}$). This was indeed observed in classic experiments from Ris [84]. However, as we will see below, this observation is also consistent with alternative models of anaphase, even ones in which forces from kinetochores do not contribute to chromatid motions. Finally, perturbing the preferred depolymerization speed of microtubules by the kinetochore (i.e., $V_{k}\to V_{k}^{\prime }$) should lead to changes in the speed of chromatids ($v_{c}\to V_{cs}+V_{k}^{\prime }$) without impacting pole motion ($v_{p}=V_{cs}$). As far as we are aware, there has not been a report of a biochemical or genetic perturbation of this nature.

#### 4.1.2. Model 2: Kinetochore- and Central Spindle-Dominated, Chromosome-Linked

Model 2: The central spindle microtubules slide apart at their preferred speed, the kinetochore causes the kinetochore microtubules and chromatids to move relative to each other at its preferred speed, and the central spindle microtubules are rigidly attached to the chromatids. While we refer to this model as “chromosome-linked”, the linkage may actually be to kinetochore microtubules very near to the kinetochore [29,49,75,76,77,78,79], which could give rise to a similar effect as a direct connection to chromatids (Figure 5B).

Assumptions:

- Dominant kinetochores: $v_{c}-v_{km}=V_{k}$ (from Equation (2))
- Dominant central spindle: $v_{cm}=V_{cs}$ (from Equation (3))
- Central spindle microtubules are linked to the chromosomes: $v_{c}=v_{cm}$
- Kinetochore microtubules are linked to the pole: $v_{p}=v_{km}$
- Astral pulling forces position the spindle, but do not alter its internal dynamics

Consequences:

- Pole movement: $v_{p}=V_{cs}-V_{k}$
- Chromatid movement: $v_{c}=V_{cs}$
- Central spindle microtubule movement: $v_{cm}=V_{cs}$
- Kinetochore microtubule movement: $v_{km}=V_{cs}-V_{k}$

In this model, chromosome segregation is driven solely by pushing from the central spindle. Forces from the kinetochore do not contribute to chromatid motion. Rather, the kinetochore draws the poles closer to the chromatids (with speed $V_{k}$) as the chromatids move apart (with speed $V_{cs}$), resulting in a net movement of the poles (with speed $V_{cs}-V_{k}$) that is slower than the speed of the chromatids.

**Figure 6:**
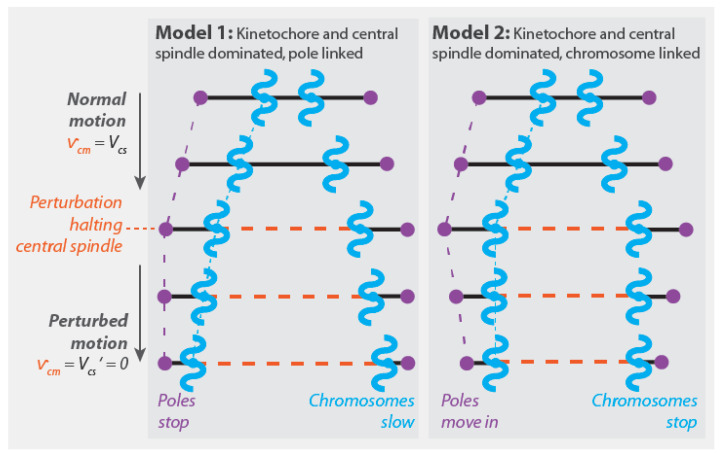
Experimental perturbations can be used to differentiate between different mechanical models of anaphase. For a perturbation that selectively halts the motion of the central spindle microtubules (i.e., by causing the preferred speed of the central spindle microtubules, $V_{cs}$, to become zero, $V_{cs}\to V_{cs}^{\prime }=0$), Models 1 and 2 (Figure 5A,B) both predict that the speed of the central spindle microtubules, $v_{cm}$, becomes zero (i.e., $v_{cm}=V_{cs}^{\prime }=0$). However, this change leads to different subsequent motions of chromosomes and poles in the two models. In Model 1, this perturbation causes spindle poles to cease moving, while chromosome motion continues (at a reduced rate). In Model 2, this perturbation causes chromosomes to cease moving. In both Models 1 and 2, this perturbation is predicted to have no impact on the relative motion of chromosomes and poles.

One intrinsic attribute of Model 2 is that it predicts that chromosomes move at the same speed as central spindle microtubules when anaphase A and anaphase B occur simultaneously ($v_{c}=v_{cm}=V_{cs}$) (Figure 5B). This has recently been observed in mitotic human tissue culture cells [50]. That study also found that damaging the central spindle with a laser led to the immediate cessation of anaphase chromosome motion, which is consistent with Model 2 (taking the laser damage to cause $V_{cs}\to V_{cs}^{\prime }=0$ gives $v_{c}\to 0$) (Figure 6). These results are both in apparent contradiction to the expectations of Model 1 (Figure 6 and Figure 5A).

In many metazoan spindles, including grasshopper spermatocytes, human tissue culture cells, and the first mitotic division of *C. elegans*, chromosome segregation can proceed even after the connection between poles and chromatids are severed by a laser or needle [50,78,85,86,87,88]. These observations are consistent with Model 2.

Model 2 predicts that perturbing the central spindle microtubules (i.e., $V_{cs}\to V_{cs}^{\prime }$) impacts pole motion ($v_{p}\to V_{cs}^{\prime }-V_{k}$) without influencing the relative movement of chromatids and poles ($v_{c}-v_{p}=V_{k}$). As noted in Section 4.1.1, this was found in classic work by Ris [84]. A key difference between Model 1 and Model 2 is that Model 2 predicts that perturbing the preferred depolymerization speed of microtubules by the kinetochore (i.e., $V_{k}\to V_{k}^{\prime }$) should result in an impact on pole motion ($v_{p}\to V_{cs}-V_{k}^{\prime }$) without changing the speed of chromatid motion ($v_{c}=V_{cs}$). In contrast, in Model 1, this same perturbation would alter the speed of chromatids without influencing pole motion. We are not aware of any published biochemical or genetic perturbation that can be used to evaluate the validity of these predictions.

#### 4.1.3. Model 3: Kinetochore- and Astral-Dominated, Pole-Linked

Model 3: The astral region generates substantial forces that pull the spindle’s poles apart; the central spindle microtubules are rigidly attached to the pole and passively respond to the forces acting on them. The kinetochore causes the kinetochore microtubules and chromatids to move relative to each other at its preferred speed (Figure 5C).

Assumptions:

- Dominant kinetochores: $v_{c}-v_{km}=V_{k}$ (from Equation (2))
- Central spindle microtubules are linked to the pole: $v_{p}=v_{cm}$
- Kinetochore microtubules are linked to the pole: $v_{p}=v_{km}$
- Subordinate central spindle mechanics that act passively ($V_{cs}=0$), with a force on central spindle microtubules of: $F_{cs}=-\eta_{cs}v_{cm}$ (from Equation (3))
- Force on poles is a balance from astral pulling force, $F_{a}$, and tension from the central spindle microtubules: $F_{a}=-F_{cs}$

Consequences:

- Pole movement: $v_{p}=\frac{F_{a}}{\eta_{cs}}$
- Chromatid movement: $v_{c}=\frac{F_{a}}{\eta_{cs}}+V_{k}$
- Central spindle microtubule movement: $v_{cm}=\frac{F_{a}}{\eta_{cs}}$
- Kinetochore microtubule movement: $v_{km}=\frac{F_{a}}{\eta_{cs}}$

In this coarse-grained model, chromosome segregation results from a sum of two processes: the poles being pulled apart by astral pulling forces (with speed $\frac{F_{a}}{\eta_{cs}}$) and the chromatids being pulled toward the poles by the kinetochore (with speed $V_{k}$).

The key distinction between Model 3 and Models 1 and 2 is that, in Model 3, pole separation results from pulling forces from the astral region. In contrast, astral pulling forces in Models 1 and 2 merely position the spindle, without contributing to its elongation. The presence of astral pulling forces has been demonstrated in diverse spindles by severing the spindle with a laser and observing a subsequent rapid motion of the spindle pole containing fragments towards the cell surface [45,46,89,90,91]. However, the extent of motion of spindle poles after laser ablation does not provide insight into whether the astral pulling forces that are present are sufficiently strong to influence the internal dynamics of the spindle, such as pole motion and chromatid motion. As described in Section 3.4.2, the velocity of a pole-containing fragment that is subject to astral pulling forces will be $v=\frac{F_{a}}{\eta_{cyto}}$, which can be quite large even for small forces, due to the small drag associated with moving spindle fragments through the cytoplasm (i.e., due to the small value of $\eta_{cyto}$). A more definitive test of the importance of pulling forces to chromatid separation, as postulated in Model 3, would be to reduce the pulling forces, by genetic perturbation or laser ablation of astral microtubules (thereby changing $F_{a}\to F_{a}^{\prime }$), and measure the subsequent change in pole motion and chromatid motion. Model 3 predicts that such a disruption will produce an equal reduction in pole motion and chromatid motion. As far as we are aware, no measurements showing this result have been reported for any spindle.

#### 4.1.4. Model 4: Central Spindle- and Astral-Dominated, Chromosome-Linked

Model 4: The astral region generates substantial forces that are sufficient to perturb the dynamics of the kinetochore, while the central spindle microtubules slide apart at their preferred speed. The central spindle microtubules are rigidly attached to the chromatids. As in Model 2, while we refer to this model as “chromosome-linked”, the linkage may actually be to kinetochore microtubules very near to the kinetochore [29,49,75,76,77,78,79], which could give rise to a similar effect as a direct connection to chromatids (Figure 5D).

Assumptions:

- Dominant central spindle: $v_{cm}=V_{cs}$ (from Equation (3))
- Central spindle microtubules are linked to the chromosomes: $v_{c}=v_{cm}$
- Kinetochore microtubules are linked to the pole: $v_{p}=v_{km}$
- Kinetochore mechanics results in a force on kinetochore microtubules of: $F_{k}=\eta_{k}\left(\left(v_{c}-v_{km}\right)-V_{k}\right)$ (from Equation (2))
- Force on poles is a balance from astral pulling force, $F_{a}$, and tension from the kinetochore microtubules: $F_{a}=-F_{k}$

Consequences:

- Pole movement: $v_{p}=V_{cs}+\frac{F_{a}}{\eta_{k}}-V_{k}$
- Chromatid movement: $v_{c}=V_{cs}$
- Central spindle microtubule movement: $v_{cm}=V_{cs}$
- Kinetochore microtubule movement: $v_{km}=V_{cs}+\frac{F_{a}}{\eta_{k}}-V_{k}$

In this coarse-grained model, chromosome segregation is driven solely by pushing from the central spindle. Forces from the astral region and kinetochores do not contribute to chromatid motion. Rather, the chromatids move apart (with speed $V_{cs}$) while the astral region pulls the poles away from the chromatids as the kinetochore pulls the pole to the chromatid.

Model 4 is similar to Model 2, except that in Model 4 astral pulling forces impact pole motions (without influencing chromatid separation). One key prediction of Model 4 is that reducing astral pulling forces ($F_{a}\to F_{a}^{\prime }$) should reduce pole movement ($v_{p}\to V_{cs}+\frac{F_{a}^{\prime }}{\eta_{k}}-V_{k}$) without impacting chromatid motion ($v_{c}=V_{cs}$). This prediction is approximately fulfilled in the first mitotic division of *C. elegans*, in which genetically perturbing astral pulling forces, via *gpr-1/2 RNAi*, leads to a large reduction in pole separation but only a very small change in chromatid motion [50]. Control spindles have very little anaphase A (consistent with $\frac{F_{a}}{\eta_{k}}\approx V_{k}$), while *gpr-1/2 RNAi* spindles exhibit substantial anaphase A and anaphase B (consistent with $\frac{F_{a}^{\prime }}{\eta_{k}}<V_{k}$). It was observed that in these perturbed cells, chromatids, and central spindle microtubules move at the same speed, even when anaphase A and anaphase B occur simultaneously, consistent with $v_{c}=$ $v_{cm}=V_{cs}$, as predicted in Model 4. In Model 4, chromosome segregation should proceed when the chromatids are no longer attached to spindle poles. Anaphase chromatid motion does continue in *C. elegans* spindles, albeit at a reduced speed, after laser ablation is used to sever the connection between the poles and chromatids (or the poles are destroyed entirely) [50,86].

#### 4.1.5. Mechanism of Chromosome Segregation in the Four Models of Anaphase

We have described four different coarse-grained mechanical models of anaphase. These models all describe spindles undergoing identical motions of chromosomes and poles. The models all contain the three force-generating regions described throughout this text: the kinetochore, the astral region, and the central spindle. However, the mechanism of chromosome segregation differs in these models. The largest distinction is between Models 1 and 3, in which the central spindle microtubules are strongly linked to the poles, and Models 2 and 4, in which the central spindle microtubules are strongly linked to the chromosomes. In the pole-linked models, chromatid motion is a sum of two processes: separation of the spindle poles (by pushing forces from the central spindle in Model 1, and by pulling forces from astral microtubules in Model 3) and movement of the chromatids to the poles (by forces from the kinetochore). In the chromosome-linked models, chromatid motion is only one process, pushing from central spindle microtubules (while pole motion results from the combination of movement of chromatids and forces from the kinetochore and the astral region).

The pole-linked and chromosome-linked models correspond to two different mechanical mechanisms of chromosome segregation. What experiments can be performed to determine which mechanism is operative in a spindle under study? Since all of the models discussed in this section can exhibit the same pattern of pole and chromosome motions, observing pole and chromosome motions cannot be used to distinguish between the mechanisms of chromosome segregation. Similarly, since all models contain the same force-generating modules, establishing the presence of those modules in a spindle cannot be used to distinguish between the mechanisms of chromosome segregation. There are three key distinguishing features between pole-linked and chromosome-linked models: (1) If anaphase A and anaphase B occur simultaneously, then chromatids will move at the same speed as central spindle microtubules in chromosome-linked spindles, while chromatids will move at a greater speed than central spindle microtubules in pole-linked spindles (compare Figure 5A,C to Figure 5B,D); (2) if anaphase A and anaphase B occur simultaneously, then perturbations that stop the motion of central spindle microtubules will stop chromatid motion in chromosome-linked spindles (Figure 6). In pole-linked spindles, such perturbations will halt pole motion while allowing chromatids to continue to separate due to forces generated by kinetochores moving chromatids to the stationary poles; and (3) in chromosome-linked spindles, perturbations that change the preferred depolymerization speed of microtubules by the kinetochore should impact pole motion without modifying the speed of chromatid separation. In contrast, in pole-linked spindles, such perturbations should reduce chromatid motion without impacting the movement of the pole.

As mentioned above, it has recently been shown that when human tissue culture cell spindles and the first mitotic spindle in *C. elegans* are simultaneously engaged in anaphase A and anaphase B: (1) chromatids move at the same speed as central spindle microtubules; and (2) damaging the central spindle leads to the complete cessation of chromatid motion [50]. These observations suggest that chromosomes are tightly linked to central spindle microtubules in those two spindles, which implies that chromosomes segregate due to pushing forces from these microtubules. Investigating the impact of perturbing the preferred depolymerization speed of microtubules by the kinetochore would further test this possibility. One practical difficulty with such experiments is that kinetochores might also be required to link chromatids to central spindle microtubules, in which case the kinetochore perturbation would only give interpretable results if that activity was maintained.

### 4.2. Additional Mechanical Models of Anaphase

The four coarse-grained mechanical models of anaphase described above are highly idealized. In reality, all of the force generators in the spindle will respond to the forces acting on them, so none of them will operate at precisely their preferred speed. In general, all force generators could have some degree of impact on each other and the motions of poles, chromosomes, and the different microtubule populations of the spindle. It is also a simplification to treat all the force-velocity relationships as linear. Furthermore, many more mechanically-distinct models of anaphase are possible, including different linkages between components and different relative strengths of force generators. The four models described above also neglect additional sites of force generation that are thought to be relevant in different anaphase spindles [3,4,5,6].

Many spindles do not contain all three of the sites of force generation described above. For example, *C. elegans* female meiotic spindles appear to lack significant forces from astral regions. These spindles can move chromosomes in anaphase without kinetochores [92], though kinetochores are required to separate homologous chromosomes [93]. At later stages of anaphase in *C. elegans* female meiotic spindles, they lack kinetochore microtubules, laser ablation between chromosomes completely stops chromosome motion, and central spindle microtubules slide apart at the same speed as chromosomes [50,94]. This suggests that chromosome segregation at these later stages of anaphase is likely driven by pushing from central spindle microtubules (though how those pushing forces are transmitted to chromosomes remains unclear). Kinetochores are also not required for anaphase chromosome motion in mouse female meiotic spindles [95] and chromosome fragments lacking kinetochores can be divided by spindles [96].

These models could be extended to attempt to represent Nicklas’ microneedle experiments in grasshopper spermatocytes. This seems to be straightforward to do by adding an applied force to chromosomes and solving for their movement with the new balance of forces. Such a calculation leads to different predictions for the source of force acting on chromosomes for the four different models discussed above. However, there is a conundrum: why did the applied force from Nicklas’ microneedle halt the motion of chromosomes relative to the rest of the spindle, instead of simply displacing the entire spindle? Presumably, the explanation is that, in those very flat cells, many microtubules on the surface of the spindle contact the cell cortex, producing forces all along the spindle. If force from the cortex are not confined to the astral region, but rather act on all classes of microtubules, then the behavior of the spindle likely cannot be captured by simple one-dimensional models like those described above. This makes it challenging to interpret the force-velocity curve that Nicklas measured (Figure 3B) in terms of forces generated by kinetochores, the astral regions, and central spindle microtubules.

## 5. Conclusions and Outlook

There are both extensive commonalities and contrasts between different spindles. A variety of force-generating sites are believed to be important in spindles, including three that have been particularly widely discussed: the kinetochore, the astral region, and the central spindle. Even when these force generators operate similarly, their contribution to anaphase motions can vary greatly, depending on how they are connected with each other. Thus, common force-generating elements of spindles can be arranged to produce contrasting mechanisms of chromosome segregation, and the mere presence of a force-generating process does not necessarily mean that it is responsible for segregation.

In this manuscript, we reviewed research on the mechanics of chromosome segregation, and organized prior results using simple coarse-grained models. While these models are highly idealized, they do respect fundamental mechanical principles, including force balance and the local nature of relevant interactions. We constructed four different coarse-grained models of spindles simultaneously undergoing anaphase A and anaphase B. While these models all contain the same three force-generating sites, they lead to different mechanisms of chromosome segregation and make different experimentally testable predictions. The most significant difference is between models in which central spindle microtubules are linked to the poles and models in which those microtubules are linked to chromosomes. In pole-linked models, chromatid motion is a sum of two processes: separation of the spindle poles (by pushing forces from the central spindle or pulling forces from the astral region) and movement of the chromatids to the poles (by forces from the kinetochore). In chromosome-linked models, chromatid motion is only one process, driven by pushing from central spindle microtubules (while pole motion results from the combination of movement of chromatids and forces from the kinetochore and the astral region).

There are still large gaps in our understanding of chromosome segregation. Additional mechanical measurements are greatly needed, as are better characterizations of the relative motions of the components of spindles in anaphase and their responses to spatiotemporally targeted physical and molecular perturbations. The models presented in Section 3 and Section 4 are merely caricatures. It is clearly an oversimplification to sharply divide spindles into those in which central spindle microtubules are pole-linked and those in which they are chromosome-linked. Central spindle microtubules are actually part of a complex network of microtubules that include kinetochore fibers, and additional non-kinetochore, non-central spindle microtubules (which are the majority microtubules in many metazoan spindles). Electron microscopy studies of metazoan spindles show that some central spindle microtubules have both their ends between segregating chromosomes, while others extend to the region between chromosomes and poles; many central spindle microtubules form bundles that split to contact multiple kinetochore fibers; many central spindle microtubules appear to associate with chromosomes [49,50]. More sophisticated coarse-grained models are needed to understand the behaviors of such networks and their implications for chromosome segregation. It will still be necessary for such models to obey the fundamental mechanical principles discussed in this review, such as force balance and the presence of only short-range forces.

“Active matter” theories seem a particularly promising basis for creating more physically realistic coarse-grained models of spindles [97,98]. From this perspective, the spindle is considered to be an orientationally ordered, viscous (or viscoelastic) material, with internally generated active stresses due to the presence of molecular motors and microtubule dynamics. It has been shown that active matter theories can quantitatively explain the morphology and internal dynamics of metaphase spindles [99,100], as well as the impact of inhibiting molecular motors [101]. Such continuum theories provide a natural framework to understand how forces (or more properly, stresses) propagate through the spindle [102,103]. Active matter theories have not yet been used to investigate the mechanics of anaphase and chromosome segregation. One challenge is to properly incorporate chromosomes into an active matter theory of spindles. It may be advantageous to explicitly model the extended nature and material properties of chromosomes, since the deformation of the chromosomes can serve as a force sensor and because the mechanics of chromosomes have been proposed to influence the spindle [104,105,106,107,108]. A multi-scale modeling approach can then be used to connect the continuum level description of the spindle to the more microscopic behavior of the molecules that constitute it. Encouraging recent advances in generic micro-to-macro theories of networks of microtubules and molecular motors [62,109,110] will have to be further extend to adequately model the specialized sites of force generation in anaphase spindles discussed in this review. If successful, such an active matter theory of anaphase would be capable of accurately representing different spindles through changes in the model’s parameters. This will surely reveal that many additional mechanically distinct mechanisms of chromosome segregation are possible.

More realistic active matter theories will likely show a range of intermediates between the extreme pole-linked and chromosome-linked models discussed in Section 4. Still, the distinction between these two limits is conceptually helpful, and the behavior of certain real spindles might be well approximated by one limit or the other. As discussed above, it seems that chromosomes are tightly coupled to central spindle microtubules in human mitotic tissue culture cells, the first mitotic division of *C. elegans*, and (at least in late anaphase) *C. elegans* meiosis. This implies that chromosome segregation in those spindles is driven by pushing from central spindle microtubules. It is tempting to hypothesize that this mechanism might apply generally to large metazoan spindles, and perhaps more broadly; but, of course, more data is needed to confirm or refute that. If correct, this would mean that force generation from kinetochores does not significantly contribute to segregating chromosomes in anaphase in many spindles. Can that be reconciled with the observation that kinetochores are universally conserved and required for accurate chromosome segregation [22,23]? We speculate that the tight coupling between central spindle microtubules and chromosomes is primarily due to central spindle microtubules binding kinetochore fibers close to the kinetochore. In this picture, the function of the central spindle would be to produce the forces that segregate chromosomes in anaphase. The function of the kinetochore would be to properly connect chromosomes to microtubules such that these forces lead to an equal division of chromosomes between the two daughter cells. This would imply that lagging chromosomes and chromosome segregation errors are likely caused by disruption in how forces are transmitted from the central spindle to chromosomes, either due to the defects in the arrangement of kinetochore fibers or defects in the linkages between kinetochore fibers and central spindle microtubules.

Claims about the “function of” the kinetochore, or any other aspect of the spindle, are only meaningful as a shorthand for evolutionary statements regarding adaptation and selection. For example, as mentioned earlier, some spindles have centrioles at their poles, while other spindles do not. Centrioles are also found at the base of cilia. While spindles are universally conserved in Eukaryotes, centrioles and cilia have been lost in many different lineages. Every lineage that loses cilia also loses centrioles, arguing that the primary function of centrioles is related to their role in cilia, not spindles [13,111,112]. Saying that “the primary function of centrioles is related to their role in cilia” is shorthand for saying that the selective advantage associated with maintaining centrioles is primarily dependent on the presence of cilia. In contrast, other variations in spindles and chromosome segregation mechanisms discussed throughout this review might not be due to adaptation [113]. Indeed, it has been proposed that many cellular and molecular aspects of organisms are largely shaped by nonadaptive processes [114,115,116].

One possibility is that selection may strongly constrain some aspects of spindles (such as those which impact chromosome segregation accuracy), while allowing others to vary. If this view is correct, then variation might be facilitated by the intrinsic redundancy of spindle mechanics: different molecular mechanisms may produce the same coarse-grained mechanical element (e.g., dynein may link kinetochore microtubules to poles in some organisms, while kinesin-14 may play that role in other organisms), and different mechanical mechanisms may produce the same motions of chromosomes (e.g., the four distinct mechanical models in Section 4 can all lead to identical movements of chromosomes and poles). Thus, the movement of chromosomes in anaphase might be conserved, even while the molecular and mechanical mechanisms responsible for those movements change. Such a scenario, called systems drift, has been proposed for the evolution of spindle movements of the first mitotic division of nematodes [117]. The relevance of this theory, and other possible explanations for the diversity of spindles and chromosome segregation mechanisms, can be most rigorously investigated using well-established methods from evolutionary genetics [118,119]. Systematic evolutionary genetic studies of spindles would help clarify why spindles display counterintuitive properties, such as being able to generate far larger forces than seems necessary to move chromosomes in anaphase.

It is still rare to apply such evolutionary approaches to cell biological processes, but a number of researchers are taking up the challenge [120,121,122]. For example, many aspects of the diversity of kinetochores can be explained by the centromere-drive model [123,124]. Centromeres are the specialized regions of DNA on each chromosome that promote the formation of kinetochores. The centromere-drive model is based on the asymmetry of the four products of female meiosis: only the one in the egg can be transmitted to the next generation, while the three in polar bodies are destined to perish. This can lead to positive selection for “selfish” genetic elements that preferentially segregate to the egg. By positing that kinetochore evolution is shaped by the resulting genetic conflicts, the centromere-drive model can explain variations in the number of microtubules that kinetochores bind and the surprisingly rapid rate of evolution of kinetochore proteins. In this view, diversity in kinetochores is driven by selection, even though the drive processes that produce this diversity leaves organisms no better off (and perhaps worse off).

In another series of studies, a combination of evolutionary genetics and biophysics was used to investigate spindle length and the elongation of the spindle in anaphase in the first mitotic division in nematodes [43,125,126]. The evolutionary genetics aspect revealed that selection acts predominantly on the size of cells, and only indirectly influences the spindle through its scaling with cell size. The biophysical aspects explained how forces from the spindle’s astral region varies with cell size, and found that this change in forces was sufficient to account for the scaling of the spindle with cell size. Taken together, these two lines of work led to a theory of spindle behaviors that makes quantitatively accurate predictions of the diversity in spindles across 100 million years of nematode evolution. These findings argue that variations in some of the aspects of spindle mechanics and behaviors discussed throughout this review might be caused by the indirect effects of selection acting on other, correlated processes.

Much work remains. A combination of biophysics and evolutionary genetics has not yet been applied to the mechanics of chromosome segregation. Once properly developed, coarse-grained models like those described above could be used to describe the diverse mechanics of chromosome segregation in different spindles, while evolutionary genetics could be used to understand the causes and functional consequences of this diversity. If successful, such a theory would reveal how and why the mechanical mechanisms of chromosome segregation work as they do.
